# Contests over reproductive resources in female roller beetles: Outcome predictors and sharing as an option

**DOI:** 10.1371/journal.pone.0182931

**Published:** 2017-08-10

**Authors:** Ivette A. Chamorro-Florescano, Mario E. Favila, Rogelio Macías-Ordóñez

**Affiliations:** 1 Facultad de Ciencias Biológicas y Agropecuarias, Universidad Veracruzana, Tuxpan, Veracruz, Mexico; 2 Red de Ecoetología, Instituto de Ecología A.C., Xalapa, Veracruz, Mexico; 3 Red de Biología Evolutiva, Instituto de Ecología A.C., Xalapa, Veracruz, Mexico; University of Vienna, AUSTRIA

## Abstract

Fights among females are frequent, although less attention has been placed on them than on male fights. They arise when females compete for food, oviposition, mates, brooding sites, or access to resources which increase offspring survival. It has been shown that the outcome of female fights may be less predictable by asymmetries in resource holding power, than in male fights. Male roller beetles fight over food resources, food balls, needed for mating and nesting, and it has been show in some species that asymmetries in reproductive experience and resource holding power in terms of size predict fight outcome, including ties in which contenders cut and split the food ball. In this study, we tested the influence of asymmetries in reproductive status (experience) and body size on female fight outcome in the carrion roller beetle *Canthon cyanellus cyanellus*. As predicted, and as previously found for males of the same species, female reproductive status of both contenders and relative size predict fight outcome. Larger and reproductively experienced contenders have a higher probability of winning. Furthermore, ties are more likely in fights involving opposing asymmetries (vgr. Large reproductively naïve owner versus small reproductively experienced intruder). Also as predicted, food ball splitting is more likely to be started by the predicted loser. This mode of resource sharing may be the result of a fighting strategy in which the costs of continuing to fight are greater than the benefits of not splitting, if a fraction of the disputed resource is more than the minimum needed for the present reproductive needs, and reduces costs associated to a longer fight.

## Introduction

Social selection is a general concept that includes social competition for resources other than mates, and sexual selection is a special case of social selection [1]. Social selection implies differential success in social competition whatever the resources at stake, conducing to differential reproductive success, which ultimately implies differential gene replication [1]. Competition for non-sexual resources may involve the same sort of traits produced by sexual selection, but selected by a socially mediated mechanism [2]. Fights among females are frequent in nature, and although less attention has been placed on them compared to male-male contests, in recent years more and more studies have searched for factors affecting their outcome [3–5]. Usually, fights arise when females compete for food resources and/or ovipositional or breeding sites which are difficult to obtain and which enable them to secure egg laying, as well as for access to resources which increase offspring survival, and may even be related to the acquisition of social status [1–2, 6–10]. The duration and outcome of female-female fights for a limited resource do not necessarily correlate with traditional measures of Resource Holding Power (RHP *sensu* Parker [11]) such as size, as it is usually the case in males, but with costly social traits such as aggression, among others [12].

The physiological status of females can be a factor influencing fight outcome. Some studies have found that the number of reproductive reduces female longevity [13–19], probably because the energy invested in reproduction is not available for tissue maintenance. This fact may increase the value of resources and thus their aggressiveness [20–23]. Following Grafen’s [24] concept of divisive asymmetries, individuals with lower reproductive life expectancy would not be selected to respect asymmetries that would put them in a loser role, because if they do, they would never have more opportunities to reproduce. Under such circumstances, only defending the resource against all rivals, no matter their condition or quality, would give these contenders their only reproductive opportunity, a condition named the “desperado effect” by Grafen [24].

Dung and carrion roller beetles are a nice group to explore factors related to intrasexual fights. Male fights for ownership of food balls and females during the reproductive period have been well described in several species of dung beetles [25–31], and recently analyzed in the context of RHP asymmetries. Male-male fights in roller beetles occur between an owner of a food ball, generally rolled with a female companion, and an intruder male that tries to steal the food ball and even the female. Males fight for food which allows the winner to obtain a mate. In *Canthon cyanellus cyanellus*, a carrion roller beetle, asymmetries in ownership and RHP during male-male contests influence the ability of the contestant to defend or gain food resources for reproduction [32–34]. Furthermore, the reproductive status of both contenders is also related to the chance of winning: previously mated owners have a higher probability of winning than virgin owners. Males of similar size tended to split the food ball, sharing the resource [33].

In *C*. *c*. *cyanellus* and in other roller beetle species, females usually assume a passive role, climbing on the food ball during rolling [6–27]. Nevertheless, females also participate in rolling and fighting for ownership of the resource against other females [33, 35]. Female fights are not for mates but for an ecological resource, the food ball used for breeding since they transform it into one or several brood balls, depending on the species [35]. Thus, in *C*. *c*.*cyanellus* and in other roller beetle species, female competition would be mostly shaped by different evolutionary forces than male competition, mostly driven by intrasexual selection.

The dynamic of female-female contests has never been analyzed in roller beetles. In this study, we experimentally analyze the effect of food ball ownership, body size and reproductive status (reproductively naïve or reproductively experienced) of female contestants on the outcome of fights in the carrion roller beetle *C*.*c*. *cyanellus*. We expect that these asymmetries influence each contender’s perceived resource value (*sensu* Enquist & Leimar [36]), resource holding power, and thus fight outcome. We predicted that large female owners of food balls should have greater probabilities of winning fights than small intruder females. When female owners are confronted with intruders of a similar size they may escalate fights, but they may also reduce the cost of scaling the aggression if they split and share the food resource. Reproductively experienced female owners should be more aggressive than reproductively naïve females, thus the latter are expected to lose fights or tend to split the resource. It is also expected that reproductively experienced females, closer to dying than naïve females and thus with more to lose, have greater probabilities of winning fights, even when they are smaller than intruder females. When small female owners confront large intruders, opposing asymmetries will tend to compensate and both contenders are expected to split the food ball. We also predicted, following Mesterton-Gibbons & Sherratt [37] that splitting will be started more often by the contender with lower probabilities of winning the full resource.

## Material and methods

Specimens of *C*. *c*. *cyanellus* were collected at the Los Tuxtlas Tropical Biology Station (18° 34' N, 95° 04' W), run by the National Autonomous University of Mexico in Veracruz, Mexico. Individuals were reared in an insectarium at 26°C ± 1°C, 70% ± 10% RH, and a photoperiod of 12 L: 12 D (following Favila [38]) to standardize the age and reproductive status of individual hatchlings. Experimental females were between 45 to 65 days old (age within this range not related to treatment), the reproductive age of this species [38]. Female’ total length was measured (from clypeus to pygidium) with a digital caliper (resolution: 0.01 mm) and weighed on a digital balance (0.002/10 g). As in a previous study conducted with males [35], three size groups were established for females: two with contrasting sizes in order to stage size-asymmetrical fights, large (7.80 to 8.20 mm) and small (6.80 to 7.20 mm), and a group of intermediate size individuals within a short size range (7.40 to 7.60 mm) to stage near-size-symmetrical fights among them. The reproductive status was either reproductively naïve (female that had never mated prior to the experiment) or reproductively experienced (females that had mated at least 5 to 7 times prior to the experiment and built at least 3 brood balls). Twelve treatments of dyadic interactions were established based on the body size and reproductive status of each female (Table 1).

**Table 1:** Dyadic interactions treatments established based on relative body size (RBS) and reproductive status (RS) of owner and intruder *C*. *c*. *cyanellus* females.

Treatments		
Owner RS/RBS	Intruder RS/RBS	N
Naïve/Smaller	Naïve/Larger	19
Naïve/Larger	Naïve/Smaller	20
Naïve/Similar	Naïve/Similar	18
Experienced/Smaller	Experienced/Larger	20
Experienced/Smaller	Experienced/Smaller	19
Experienced/Similar	Experienced/Similar	20
Naïve/Smaller	Experienced/Larger	22
Naïve/Larger	Experienced/Smaller	23
Naïve/Similar	Experienced/Similar	20
Experienced/Smaller	Naïve/Larger	17
Experienced/Larger	Naïve/Smaller	19
Experienced/Similar	Naïve/Similar	21

The females were marked with a small spot of metallic ink on the elytra or on the pronotum to identify each contender [39]. In each trial a female was introduced into an observation arena (150 x 15 mm Petri dish with filter paper) containing an artificial ground beef food ball (0.8 g). Females that rolled the food ball at least 15 minutes were considered to be owners [33]. Then, an intruder female was introduced into the observation arena, starting the observation. The female that kept the food ball in its possession for at least 10 minutes, while simultaneously preventing the approach of the intruder, was considered to be the winner. When contenders split the food ball and both obtained a part, the result was considered a tie. In six trials the intruder did not try to steal or split the food ball after 90 minutes and the original owner was also considered the winner when (excluding these trials of the analysis did not significantly change the results, so we kept them in the analysis) [33].

### Statistical analysis

All statistical analyses were carried out with the statistical program R version 3.1.0. [40]. As null hypothesis, the random probability for each of the three potential outcomes was assigned as 0.33. The random distribution of probabilities for each potential outcome for the owner (winning, splitting a food ball or losing it) was obtained after 5,000 randomizations ran per experiment using a previously developed R code [41] (S1 File). The overall frequency of observed events of each outcome was compared to the appropriate distribution to determine if the probability of obtaining the corresponding observed frequency was significantly different from what would be expected from a random process.

The outcome for a female owner could take one of three different states: winning (the whole food ball was retained by the owner), splitting (the food resource was divided between the combatants), or losing (the intruder obtained the whole food ball). Thus, a polytomous logistic regression [41] was used to evaluate the effect of the following independent variables of the intrasexual fight on the categorical response: owner reproductive status, intruder reproductive status and the body size of the owner relative to the contender (larger than, similar or smaller than). The second-degree interactions of the independent variables in the model were also tested. The Akaike information criterion (AIC) was used to determine the most parsimonious models. We used the *Effect Displays* R package [42], which includes functions to predict event probabilities and corresponding ± 95% confidence intervals of winning, splitting and losing [43]. Confidence intervals were used as post hoc tests. S2 File.

Finally, using the same independent variables, a logistic regression was conducted using the cbind function in R [44], on the odds ratio between tied fights in which splitting was started by the owner, and tied fights in which splitting was started by the intruder. Since the replicates of each treatment were used to build these proportions, we could not include the statistical interaction in this analysis. S4 File.

## Results

The three observed frequencies for each of the three possible outcomes were significantly different from what would be expected from a random process. Owner females won significantly more fights than expected by chance (n = 110 of 242, 0.45, P < 0.001); females split the food ball fewer times than expected by chance (n = 67, 0.27, P = 0.017); and owners lost significantly less fights than expected by chance (n = 65, 0.28, P = 0.035).

The minimum model of logistic regression showed that the predictors which significantly influenced the outcome of intrasexual fights between females were the interaction between owner reproductive status and body size asymmetry, as well as the reproductive status of both contenders and the body size asymmetry (Table 2).

**Table 2:** Polytomous logistic regression minimal model (LR Chisq likelihood ratio chi square) of the effects of relative body size (RBS: large-small, small-large and size-matched) and reproductive status of the owner and the intruder (RS: experienced and naïve) on the outcome of fights between *C*. *c*. *cyanellus* females.

	LR Chisq	Df	P
**Owner RS**	8.9941	1	0.0027
**Intruder RS**	7.7379	1	0.0054
**RBS**	29.6558	2	<0.001
**Owner RS: RBS**	13.2823	2	0.0013

**Fig 1:**
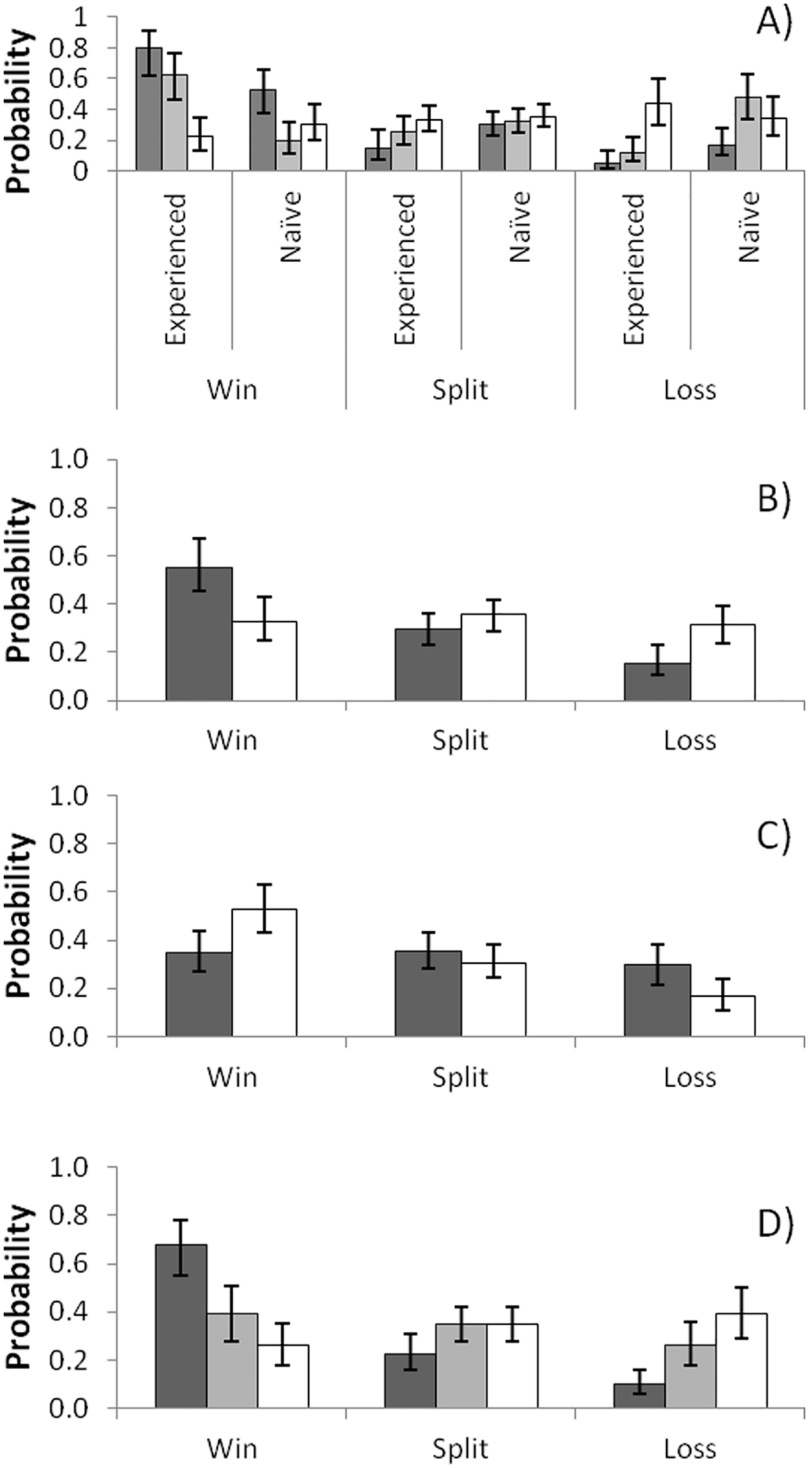
Estimated probabilities on the outcomes of fights between females of *C*. *c*. *cyanellus*. (A) Effect of the interaction between owner reproductive status asymmetry and relative body size (owner larger than intruder in black, size-matched in gray and owner smaller than intruder in white); (B) owner reproductive status (eexperiencedin gray and naïve in white); (C) effect of intruder reproductive status (experienced in gray and naïve in white); and (D) effect of relative body size (owner larger than intruder in black, size-matched in gray and owner smaller than intruder in white). Probabilities and 95% confidence intervals (whiskers) were estimated using a logistic polytomous model.

Regarding the significant factor interactions, reproductively experienced owners that were larger than or similar in size to the intruder had a significantly higher probability of winning contests than smaller experienced owner females. Large reproductively naïve owners had a significantly higher probability of winning contests than when they were size-matched, but the probability of winning for small naïve owners was not significantly different from the probability of winning for large and size-matched naïve owners (Fig 1A). Both of these results are mirrored in the corresponding probabilities of losing. Experienced and naïve owner females had a non significantly different probability of splitting the food ball in their respective groups, but large experienced owners had a significantly lower probability of splitting the food ball than small naïve owner females.

Regarding the significant effects of single factors, reproductively experienced owner females had a significantly higher probability of winning contests than losing and splitting the food ball, but there was no significant difference in the probability of splitting and losing contests (Fig 1B). Reproductively experienced owner females had a significantly higher probability of winning contests than naïve owner females. Reproductively naïve owner females had non significantly different probabilities of winning, losing or splitting the food ball during contests.

Outcome frequencies of fights between owner females and experienced intruders were not significantly different from random. However, when owner females faced naïve intruders, they had a significantly higher probability of winning than of losing or splitting the food ball (Fig 1C).

Owners that were larger than intruders had a significantly higher probability of winning than splitting or losing the food ball, but these females had non significantly different probabilities of losing and splitting the food ball (Fig 1D). Owners that were similar in size and smaller than intruders had non significantly different probabilities of wining, losing or splitting the food ball; however, larger owners had a significantly lower probability of losing contests than smaller and size-matched owners (Fig 1D).

**Fig 2:**
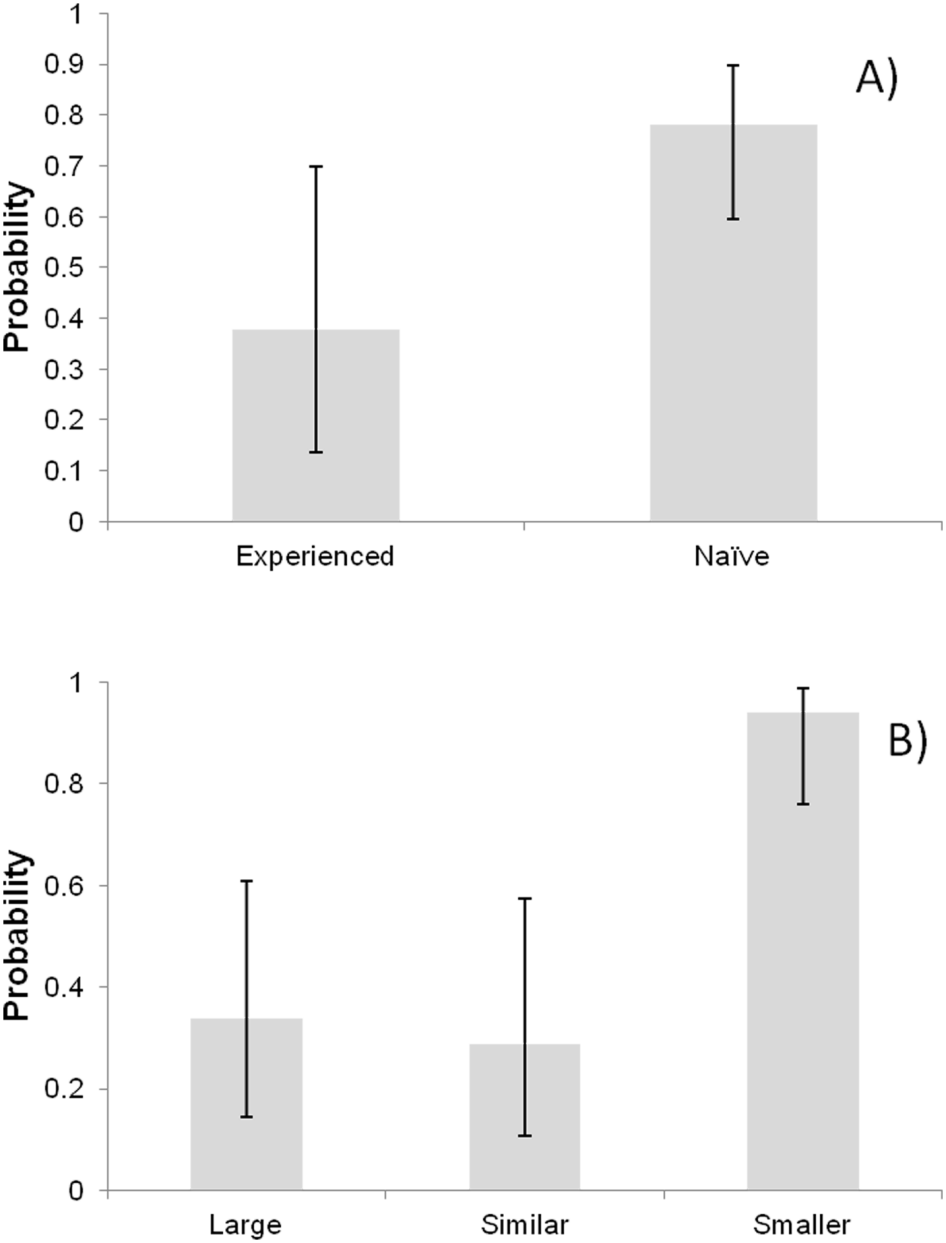
Estimated probabilities of being the contender that started splitting the food ball on tied contests. (A) Effect of owner reproductive status; (B) effect of relative body size. Probabilities and 95% confidence intervals (whiskers) were estimated using a logistic model on the odds ratio between number of intruder and owners females of *C*. *c*. *cyanellus* that started splitting the food ball. Notice that even though the model reports a significant P value for the effect of owner reproductive status, the confidence intervals overlap. This is due to the fact that although non-overlapping confidence intervals do imply a significant effect, overlapping confidence intervals do not necessarily imply a non-significant effect (see [45]).

In the case of the proportion of the 67 tied contests in which splitting was started by either the owner or the intruder, the minimum model of logistic regression showed that the predictors which significantly influenced this variable were the reproductive status of owners and the body size asymmetry (Table 3). Naïve female owners had a significantly higher probability of being the splitter of the food ball than experienced owners (Fig 2A). Female owners that were larger than intruders were not significantly different from those size-matched in their probability of being the contender that started splitting the food ball. However, female owners that were smaller than intruders had significantly higher probabilities than both of the previous two relative-size categories of being the contender that started splitting the food ball (Fig 2B).

**Table 3:** Logistic regression (LR Chisq likelihood ratio chi square) of the effects of relative body size (RBS: large-small, small-large and size-matched) and reproductive status of the owner and the intruder (RS: experienced and naïve) on the probability of being the contender that started splitting the food ball in tied fights between *C*. *c*. *cyanellus* females.

	LR Chisq	Df	P
**Owner RS**	5.0662	1	0.0244
**Intruder RS**	2.8601	1	0.0908
**RBS**	23.2595	2	<0.001

## Discussion

Ownership of a limited and greatly valued resource may generate one or many asymmetries, giving it a strategic advantage to the individual who owns the resource [36]. The owner may have greater knowledge of the resource value than the intruder, perceive him or herself as owner, and/or have higher RHP than the population average [36, 46]. In several species, fights among females for reproductive resources such as egg laying substrate may be similar to those among males of the same species [20, 47–57].

Fights among male roller beetles for the ownership of a food ball during rolling and nesting have been widely documented [26–27, 33–34], showing that ownership of the food ball, together with size (RHP) asymmetries, predict fight outcome. Our results show that food ball female owners of *C*.*c*. *cyanellus* also had a greater probability of winning fights against intruders who tried to steal the resource. The results may be similar in both sexes, but the value of the contested resources may be different for each sex. Males fight for a food ball to obtain a mate, females fight for a reproductive food resource.

In both sexes of several animal species, differences among contestants in body size result in RHP asymmetries linked to success in fights for limited resources. We found that body size influenced fighting success among females of *C*.*c*. *cyanellus*. Large females won more fights against small opponents, as is the case for males of this species [33]. Nevertheless, in females, body size may also be correlated with fecundity, which may also result in an asymmetry of perceived resource value [1, 58–60].

Another asymmetry, which influenced fight outcome among female *C*. *c*. *cyanellus* was their reproductive status. Reproductively experienced females had a greater probability of winning the resource when confronted with reproductively naïve females. In males of this species we previously found that male reproductive status modulates fight outcome. Experienced male owners confronting naïve intruders, either alone or rolling with a female, have a greater probability of winning and keeping the food ball and the female than naïve owners who confront experienced intruders [33]. In the case of females however, it has been shown that there is a decrease in the longevity of those who reproduce [38], due to the significant energy investment in mating and egg laying, which cannot be allocated to tissue maintenance. In this species, reproduction only lasts during the rainy season from May to September, therefore sexually active beetles have only a restricted time to reproduce. This is more restricted in the case of females since they remain guarding the nest balls until offspring emergence (25 to 30 days); compromising the number of nesting events that can be accomplished in one season, while males abandon the nest once laying is over (approximately 10 days after first mating) [35–36, 38]. This may explain why experienced females have a higher probability of winning a fight if this difference results in an asymmetry in perceived resource value, and thus in a more escalated fighting strategy. For males of this species, the effect that previous reproductive experience exerts on the composition of cuticular hydrocarbures can be detected by mates and opponents as well [61], probably as a measure of the contender´s competitive ability, and influence contest outcome. It is unclear whether this occurs in females and thus should be explored in future studies.

We found that the reproductive status of female *C*. *c*. *cyanellus* may play as an advantage in the outcome of fights over food balls needed for nesting, even against body size asymmetries. As predicted, asymmetries in both body size and reproductive status had an important effect on fight outcome. Furthermore, our prediction that experienced owner females, when smaller than the intruder, had greater probabilities of winning fights was corroborated. Smaller experienced females may show a decrease in reproductive opportunities thus triggering a desperado strategy during fights, defeating larger but naïve females, which probably play a more conservative fighting strategy. As stated before, this difference in perceived resource value may be a divisive asymmetry (*sensu* Grafen [24]), since experienced females are not expected to give up if the disputed resource may be their last chance of a final reproductive event.

Food sharing has been observed in different animal species [62–65]. Even when both contenders play the same strategy, there usually are asymmetries increasing the probability that one of the contenders will keep all or most of the resource, but sharing remains a likely ESS in such circumstances and the stronger opponent should be less willing to divide the contested resource [37]. Males of *C*. *c*. *cyanellus* frequently, but not always, divided food balls when both contenders were size-matched, and also when they had opposing asymmetries in terms of reproductive status and size [33]. In the case of females, food ball splitting also occurred when both contenders were size-matched, and when the owners were smaller or larger than naïve intruders, probably as a result of some form of asymmetry compensation. In these fights, females that ended up splitting the food ball and thus sharing the resource were more frequently naïve owners. Food sharing reduces energy drains and injury risks involved in sustaining a fight with an uncertain all-or-nothing outcome, and may result in a better outcome for both players and thus become evolutionary stable. In both male-male and female-female contests, food splitting seemed to occur generally after fighting, not peacefully as in other species [66, 46]. One of the contenders may, during the interaction, start dividing the food ball even if not supported by the other, which may eventually accept taking a fraction of the disputed resource instead of continuing the fight. Dubois and Giraldeau [24, 21] suggested that if the “finder” (owner) is able to adjust its behavior to match that of its opponent, sharing the food might be the best strategy for both the finder and the “joiner” (intruder). As predicted by Mesterton-Gibbons and Sherratt [37], we found that individuals more likely to lose a fight (naïve and small owners) were more often the contender that started splitting the food ball in fights that ended up in a tie, that is, sharing the food ball. In such cases, the likely winner may be forced to “accept” this outcome, especially if splitting the resource makes fighting for both pieces more difficult than when fighting for a single one, and one piece is enough to produce a nest anyway.

Since the reproductive behavior of *C*. *cyanellus* develops around the food ball needed to obtain mates (males) or produce brood balls (females)[67, 38, 35], splitting is adaptive for both contenders if both end up keeping the minimal amount of resources to nest. With a food ball rolled by a male, a female is usually able to make from two to six brood balls, and the size of a brood ball in this species seems to be more or less constant [35]. Thus the amount of food obtained by each female when they split a food ball usually should allow each female to make two or even more brood balls. In other words, the value of each of the two fractions of the food ball may be worth more than half the value of the whole food ball, *sensu* Mesterton-Gibbons and Sherratt [37], if the whole ball would also be used for one reproductive event anyway, which would not produce more than twice the number of offspring.

Several studies in roller beetles frequently gave females a passive role during rolling [25–26]. Nevertheless, our study shows that females of *C*. *c*. *cyanellus* fight for a vital reproductive resource, the food ball from which females build brood balls to nest. The outcome of fights among females is influenced by the same factors previously described for males in this species. Nevertheless, selective pressures and the actual asymmetries behind these patterns in each sex seem to be different. For males, selection to increase number of mates or paternity likelihood may result in fighting strategies similar to those that in the case of females result from selection for strategies that increase the likelihood of obtaining enough reproductive resources to make brood balls. However, females occasionally also fight for males. Females can participate in fights among males, collaborating with either the owner or even the intruder to expel the other contestant. They can also actively attack and expel one of the contestants, generally a reproductively naive male [33]. Further experiments are required to evaluate which factors are related to female aggression on males not accepted for reproduction.

Since all roller and other beetles that require dung or carrion to reproduce share the similar needs and thus the potential for intrasexual fights in both sexes, it is relevant to review what is known in different species to gain a more complete view on the effect of different factors on intrasexual fights as mechanisms of social selection. *Onthophagus sagittarius* is a tunneler dung beetle species in which females have horns related to competition for food resource for reproduction [58]. *Canthon cyanellus* and other species of dung roller beetles do not show apparent sexual dimorphism, but both sexes engage in fights using structures involved in rolling such as the *clipeous*, the tip of the head used to cut the material for food balls and to raise and push opponents, or their strong legs used to push their bodies backward when rolling (e.g. *Scarabeus laevistriatus* [68], *Canthon indigaceus chevrolati* [29], *Kepher platynotus* [30]). The fact that both sexes may engage in rolling and fighting may explain why no sexually dimorphic characters have evolved in either sex (see [69]). The comparative study of fights among females in roller dung beetles species seems a promising field for understanding the evolution of social selection in insects and other organisms.

## Supporting information

S1 File R script to estimate random distribution of probabilities for each potential outcome for owner. (R)

S2 File R script polytomous logistic regression to evaluate the effect of independent variables on fight outcome. (R)

S3 File Tab separated text data file containing variables used by S2. (TXT)

S4 File R script logistic regression on odds ratio between splitting started by owner and intruder. (R)

S5 File Tab separated text data file containing variables used by S4. (TXT)
